# Evaluation of the Diagnostic Accuracy of a New Dengue IgA Capture Assay (Platelia Dengue IgA Capture, Bio-Rad) for Dengue Infection Detection

**DOI:** 10.1371/journal.pntd.0003596

**Published:** 2015-03-24

**Authors:** Sophie De Decker, Muriel Vray, Viridiana Sistek, Bhety Labeau, Antoine Enfissi, Dominique Rousset, Séverine Matheus

**Affiliations:** 1 Institut Pasteur, Laboratoire de Virologie, Cayenne, Guyane Française; 2 Institut Pasteur, Epidémiologie des Maladies Emergentes, Paris, France; University of Pittsburgh, UNITED STATES

## Abstract

Considering the short lifetime of IgA antibodies in serum and the key advantages of antibody detection ELISAs in terms of sensitivity and specificity, Bio-Rad has just developed a new ELISA test based on the detection of specific anti-dengue IgA. This study has been carried out to assess the performance of this Platelia Dengue IgA Capture assay for dengue infection detection. A total of 184 well-characterized samples provided by the French Guiana NRC sera collection (Laboratory of Virology, Institut Pasteur in French Guiana) were selected among samples collected between 2002 and 2013 from patients exhibiting a dengue-like syndrome. A first group included 134 sera from confirmed dengue-infected patients, and a second included 50 sera from non-dengue infected patients, all collected between day 3 and day 15 after the onset of fever. Dengue infection diagnoses were all confirmed using reference assays by direct virological identification using RT-PCR or virus culture on acute sera samples or on paired acute-phase sera samples of selected convalescent sera. This study revealed: i) a good overall sensitivity and specificity of the IgA index test, i.e., 93% and 88% respectively, indicating its good correlation to acute dengue diagnosis; and ii) a good concordance with the Panbio IgM capture ELISA. Because of the shorter persistence of dengue virus-specific IgA than IgM, these results underlined the relevance of this new test, which could significantly improve dengue diagnosis accuracy, especially in countries where dengue virus is (hyper-) endemic. It would allow for additional refinement of dengue diagnostic strategy.

## Author Summary

Dengue disease has become a major global public health concern, but an ideal diagnostic test that permits early and rapid diagnosis is not yet available. Improving diagnostic performance in this area is a major challenge and necessitates the development and evaluation of new efficient, accurate methods. According to the kinetics of dengue infection in serum, virus isolation and nucleic acid or antigen detection are the most specific diagnostic methods during the early acute stage of disease; serology is often used for diagnosis later in the course of infection. In order to provide an earlier and reliable dating of the dengue infection, few recent studies showed that the detection of specific IgA in the serum is a useful diagnostic parameter. Exploring that new approach, this study was carried out to assess the performance of a Platelia Dengue IgA Capture assay for dengue infection detection, newly developed by Bio-Rad, using 184 well-characterized samples provided by the French Guiana NRC sera collection of the Institut Pasteur in French Guiana. This study revealed good overall performances of this test, constituting promising assistance in dengue diagnosis, especially in hyper-endemic countries.

## Introduction

Caused by any of four dengue virus serotypes (i.e. DENV-1 to DENV-4), dengue infection is currently the most significant arthropod-borne viral disease [1]. Whereas the World Health Organization (WHO) estimated in 2009 that 50–100 million infections occur each year [1], a recent study estimated that 390 million dengue infections occurred annually, of which more than 90 million were symptomatic [2]. World Health Organization has identified approximately 100 tropical and sub-tropical countries around the world where populations experience a high risk of dengue exposure. Because of its rapid spread and its impacts on human health, dengue epidemic has become a major and global public health concern.

Without specific therapeutics or vaccines, dealing effectively with this disease emergency requires innovative and appropriate diagnostic tools [3–6]. During the acute phase of the disease, dengue diagnosis is based on direct viral detection targeting the genome, especially by RT-PCR approaches, virus isolation on culture cell, or a viral antigen, the non-structural protein 1 (NS1) by ELISA or rapid tests. Indeed, the virus and soluble NS1 circulate in patients’ blood and persist for 5–7 days after fever onset. Indirect methods of dengue diagnosis are based on dengue-specific antibody detection, particularly the specific immunoglobulin M (IgM) by an IgM antibody-capture enzyme-linked immunoabsorbent assay (MAC ELISA), but also virus-specific IgG and IgA. Different commercial kits are available to detect these specific antibodies [1,3,5,7].

In response to the disease, IgM could be detected in 50% of cases early after infection (within 3–4 days after fever onset) and the majority of infected individuals become positive for IgM by day 5–6. IgM have been described as persisting until about 3 to 8 months post onset [7]. As for IgM, IgG are generally detectable at the end of the first week of illness (within 7–10 days after fever onset), and still detectable in serum after several months, and probably even for life. Concerning anti-dengue IgA antibodies kinetics, their positive detection often occurs one day after the beginning of the IgM time frame (on average at 5.5 days after the onset of fever) reaching their highest titres around day 8 following this onset. The IgA titre decreases rapidly until it reaches undetectable levels by day 40, indicating a shorter persistence of dengue virus-specific IgA in serum than IgM and IgG [6–11].

The dengue virus and circulating antibodies displayed well-known dynamic patterns: these patterns are influenced by the infecting serotype and patients’ clinical status, which shows significant differences following a primary or secondary dengue infection [1,7,11]. In secondary infection, IgM antibodies appear earlier or within the same time frame, but are usually at lower titres than during primary infection. They may even be undetectable. During secondary infection, the dominant antibodies are IgG, present from the previous infection and detectable at high levels, even in acute-phase serum samples. Concerning IgA dynamics in secondary infection, these antibodies in serum appeared to be slowly increasing during the first days, until reaching a higher level than in primary dengue infections [8,11].

Notably because both IgM and IgG antibodies persist for several months or years after infection, an IgM or IgG positive result from one serum sample is no more than suggestive: only seroconversion from paired serum samples-or a four-fold IgG titre increase- can confirm a recent dengue diagnosis [1]. But in practice, due to difficulties in obtaining blood samples taken on two occasions with an interval of no less than fifteen days apart, the serological analysis is carried out from a single acute-phase serum specimen and therefore provides only probabilistic diagnosis. The interpretation of these indirect tests is especially difficult in countries where dengue virus is hyperendemic and where other flaviviruses circulate, which could induce serological cross reactivity [1].

In this type of epidemiological context, obvious ways to overcome these diagnostic issues are to combine several diagnostic tests based on different approaches (direct and indirect, or multiple indirect tests) and to develop new tests targeting new infection markers. IgA thus appears to be an early, high-quality serological marker. Because dengue-specific IgA antibodies are detectable in acute-phase serum and persist for a shorter period of time than dengue-specific IgM, some studies have recognized the value of IgA detection in sera for dengue virus diagnosis using ELISA and immunofluorescence assays [7,9,12–14]. Helping to narrow the time frame of marker detection after infection, the IgA based method could be a more informative diagnostic tool and a better marker of recent dengue infection [8,10–13].

In keeping with these aims, a novel ELISA test for the detection of anti-dengue virus IgA from human sera, Platelia Dengue IgA Capture, was recently developed by Bio-Rad. The main objective of our study was to evaluate the performance of this serological dengue diagnostic test, based on specific IgA detection in clinical samples from patients exhibiting a dengue-like syndrome. Dengue diagnosis was confirmed using reference assays (detection of DENV RNA by RT-PCR and virus culture). Accuracy was also evaluated in relation to both DENV infecting serotype and DENV immune status. The second objective was to compare the Platelia Dengue IgA Capture performance to the PanBio Capture IgM test for dengue diagnosis in serum.

## Methods

### Study setting and patients sampling criteria

A total of 184 human sera were used for this study in order to reach a precision of 5% of the performance of the index test and to conform the STARD requirements [15]. The index test, Platelia Dengue IgA Capture, was evaluated using dengue diagnostic reference assays, as described below. Sera were provided from the French Guiana NRC (National Reference Center) sera collection (Laboratory of Virology, Institut Pasteur in French Guiana), stored at -80°C. The collection encompasses sera collected between 2002 and 2013 from patients exhibiting a dengue-like syndrome (fever, arthralgia, headache and/or myalgia). These sera were collected either for diagnostic purposes or for identifying the DENV serotype from patient sera already found positive for NS1 antigen in the context of epidemiological surveillance.

The 184 sera selected for this evaluation included two groups: a group of 134 sera from confirmed dengue-infected patients and a second group of 50 sera from non-dengue infected patients. Sera were classified according to the onset of fever (day 0 was defined as sera collected within 24h after the onset of fever).

A patient with febrile illness consistent with dengue fever was defined positive for DENV infection if an acute-phase serum sample was found positive for either RT-PCR targeting viral RNA [16] and/or viral isolation in *Aedes pseudoscutellaris* cell (AP61) [17]. Dengue-positive samples were selected to achieve a balanced collection of sera sampled between the third and the fifteen day following the onset of fever and of sera infected by the four DENV serotypes. All dengue-positive patients constitute the “dengue group”.

A patient with febrile illness consistent with dengue fever was defined negative for DENV infection if at least two of the three following analyses were obtained: (i) Negative RT-PCR or viral isolation from samples collected on day 0 and day 5 of the disease [16,17]; (ii) Negative NS1 detection of sera obtained prior to five days (Platelia Dengue NS1 AG, Bio-Rad); (iii) Negative in-house IgM capture assay on no less than 8 days sera [10]. All dengue-negative patients constitute the “non-dengue group”.

Convalescent serum was included in this evaluation, provided that its own paired acute-phase serum sample was available. Finally, all sera were analyzed for the presence of anti-dengue IgM and IgG using the Dengue IgM and Dengue IgG capture assay from PanBio (Panbio Dengue IgM Capture ELISA, Panbio Dengue IgG Capture ELISA—Australia), conducted according to the manufacturer’s instructions.

### Ethical considerations

No research-specific blood collection was performed for the study purpose. All the analyzed samples were remaining samples resulting from diagnosis procedures following blood collection required by the care for any patient presenting dengue-like symptoms in French Guiana, and kept by the NRC biobank for both health and scientific purposes. According to the French legislation (article L.1211–2 and related of the French Public Health Code—FPHC), biobanking and secondary use for scientific purpose of remaining human clinical samples are possible as long as the corresponding patients (or their parents if less than 18 years of age) were previously informed and had given no oral objection (documented in the medical or laboratory files) to them. Whenever no information are available concerning patient’s objection, a waiver from one of the 39 French Ethical Committees (Comités de Protection des Personnes—CPP) could be sought according to the FPHC. In the present research, those two requirements are fulfilled. Information had been given to patients through the brochure entitled “Information for patients” during the prospective blood collection, and no immediate or delayed patient’s opposition was reported by the clinicians to the Arboviruses NRC. Study ethical approval and information waiver for retrospective remaining samples were obtained from the CPP Sud-Ouest Outre-Mer III (CPP # DC 2013/27). Moreover, in application of French legislation (article L.1243–3 and related of the FPHC), the NRC biobank for research purpose had been declared to both the French Ministry for Research and the CPP Ile de France I (declaration #2010/1223). The NRC database was declared to the French Data Protection Agency (Commission Nationale de l’Informatique et des Libertés, CNIL # 1248768) and provided clinical information about the age and sex of each patient, the date of serum collection and the date on which symptoms began.

### Serum characterization

#### Virus culture and detection of DENV RNA by RT-PCR

Dengue virus infection was diagnosed virologically, based on the acute phase serum samples, by means of culture from AP61 mosquito cells [17] or reverse transcription-PCR [16].

Briefly, concerning virus isolation, acute phase serum samples were 10-fold diluted in Leibowitz medium containing 3% fetal calf serum, and dilutions were inoculated onto monolayered subconfluent AP61 cells. After seven days of culture, cells were harvested, and dengue subtypes viruses were identified by indirect immunofluorescence assay (IFA) using monoclonal antibodies specific for DENV-1,-2,-3,-4 virus (provided by CDC, Fort Collins, CO, USA).

About the DENV RNA detection by RT-PCR, total viral RNA was extracted from a 140μL sera using QIAmp Viral Minikit (Qiagen) according to the manufacturer’s recommendations. Then, 10 μL of RNA were both retro-transcripted and first-round amplified with a highly conserved primer pair, i.e. D1 (forward) and D2 (reverse) using the OneStep RT-PCR kit (Qiagen). Dengue virus sequences were amplified and typed using second-round amplification with primer D1 and four serotype-specific primers (TS1, TS2, TS3, and TS4) using the GoTaq Flexi DNA polymerase from Promega.

#### NS1 detection and serological analysis

The DENV NS1 antigen was detected using the commercial Platelia Dengue NS1 AG (Bio-Rad) according to manufacturer's instructions [18]. Finally, detection of specific dengue IgM was carried out using an in-house MAC ELISA test, as described by Talarmin [10].

### Index test principle, the Platelia Dengue IgA Capture assay

Platelia Dengue IgA Capture (Bio-Rad Laboratories—Marnes La Coquette, France) is a microplate immunoassay using immuno-capture format for detection of specific IgA against DENV in human serum or plasma. Intra- and inter-assay repeatabilities were already assessed using 4 samples, tested in the same assay in 32 replicates: coefficients of variation ranged from 2.3% for the medium positive samples to 26.8% for the low negative one (cf. the analytical and clinical performance report from Bio-Rad). The test was used strictly following the instructions provided by the manufacturer. Briefly, 200 μl of 1/100 diluted sera of each 184 patients were distributed in each well then incubated for 1 h at 37°C. The plate was then washed four times and 200 μl of conjugate were added and incubated for 1 h at 37°C. After a 4-time washing step, revelation was carried out with a TMB substrate solution for 30 min at room temperature then stopped with 1N sulfuric acid. Optical densities (OD) were read at 450/620 nm using a plate reader within 30 minutes after stopping the reaction. Results were expressed in ratio = (OD of tested sample) / (appropriate Cut-Off). Results were interpreted as positive, negative or equivocal using the ratio provided with the kit: positive result when ratio was greater than 1, negative lower than 0.8 and equivocal between 0.8 and 1.

### Panbio Dengue IgM and IgG detection and host immune status definition

Detections of specific dengue IgM and IgG were carried out using the Panbio Dengue IgM Capture ELISA and the Panbio Dengue IgG Capture ELISA kits according to manufacturer’s recommendations. An IgM/IgG ratio was used to distinguish between the primary and the secondary dengue virus infections on serum samples showing positive IgM and positive IgG results, as recommended by the World Health Organization [1]. Using patient’s sera at 1/100 dilution, dengue infections were classified as primary if the IgM/IgG OD ratio was greater than 1.2 and as secondary if that ratio was lower than 1.2. In addition, a host immune status was defined in the acute sample as being due to a primary infection when it was found positive for IgM and negative for IgG, and due to a secondary infection when it was found negative for IgM and positive for IgG. Others equivocal results or combinations were defined as unclassifiable.

### Statistical considerations

All serum analyses were tested blinded to the confirmed dengue samples (virus isolation and/or RT-PCR positive). Continuous variables were expressed as median and interquartile ranges (IQ1-IQ3) or mean ± SD and categorical variables as percentages. Differences among percentages were analyzed using the Fisher’s exact test and differences among continuous variables were analyzed using the Kruskal-Wallis test. In case of global significant differences between the groups, Bonferroni correction was applied.

AUROCs were calculated with 95% confidence intervals for Platelia Dengue IgA Capture and Panbio Dengue IgM Capture ELISA and compared using the non parametric Delong test [19,20]. Kappa (K) coefficient was calculated to evaluate the concordance between Platelia Dengue IgA Capture and Panbio Dengue IgM Capture ELISA results, using the interpretation scale of Landis-Koch [21]. Results were considered statistically significant when p<0.05. The sensitivity and the specificity for the assays were calculated based on confirmed dengue with the binomial exact 95% CIs. STATA 12.2 (StataCorp, College Station, Texas) software was used for all statistical analyses.

## Results

### Samples characteristics

One hundred and eighty-four patients presenting a dengue-like syndrome were included to estimate the Platelia Dengue IgA Capture performances. Gender (94 females, 51.1%; 90 males, 48.9%) was equally distributed between both groups (p = 0.120) (Table 1). The mean patient age was 35.8 ± 17.6 years, with age ranging from 1 to 90 years old. Mean age of the dengue positive patient group (dengue group) was 34.8 ± 1.4 years; mean age of dengue negative patient group (non-dengue group) was 36.5 ± 2.9 years, with no statistical difference between the two groups (p = 0.58).

**Table 1:** Description of serum samples (n = 184) used for evaluating the performance of the Platelia Dengue IgA Capture test according to the DENV serotype and the number of days after fever onset.

	Dengue group					Non-dengue group
Days after fever onset	DENV-1	DENV-2	DENV-3	DENV-4	Total	Total
**3**	2	2	0	2	**6**	**1**
**4**	8	1	0	1	**10**	**3**
**5**	4	2	3	3	**12**	**3**
**6**	7	4	4	4	**19**	**4**
**7**	8	3	3	3	**17**	**3**
**8**	7	2	4	3	**16**	**9**
**9**	3	2	2	1	**8**	**7**
**10**	1	5	3	2	**11**	**9**
**11**	2	2	2	3	**9**	**3**
**12**	0	1	2	1	**4**	**2**
**13**	1	1	2	1	**5**	**3**
**14**	1	1	4	1	**7**	**2**
**15**	2	1	7	0	**10**	**1**
**Total**	**46**	**27**	**36**	**25**	**134**	**50**

Sera were collected between the 3^rd^ and the 15^th^ day following the onset of fever (Table 1). The median (Interquartile 1-Interquartile 3, IQ1-IQ3) number of days between onset of fever and sample collection is 9 (7–10) and 8 (6–11) after the onset of fever for the non-dengue group and the dengue group, respectively.

### Assessment of the accuracy of the Platelia Dengue IgA Capture assay

Only one sample out the 184 tested sera was found equivocal, which represents only 0.54% of inconclusive results (Table 2). Out of the 134 dengue group sera, the IgA index assay detected 124 positive, indicating a sensitivity of 93% (95% CI, 87% to 96%). Six sera out of the 50 non-dengue group ones were also found IgA positive, demonstrating a specificity of 88% (95% CI, 75% to 95%). Out of the 10 IgA seronegative sera from dengue positive patients, 8 were collected 3–4 days after the onset of fever, with the other 2 collected 6 days following fever onset. Seropositivities for IgA broadly increased from 50% for sera collected 3–4 days after the onset of fever to 100% for those collected seven days after.

**Table 2:** Estimated performances of Platelia Dengue IgA Capture assay.

Dengue diagnosis			
Platelia Dengue IgA	Dengue group	Non-dengue group	Total
**Positive**	124	6	**130**
**Negative**	10	43	**53**
**Equivocal**	0	1	**1**
**Total**	**134**	**50**	**184**

#### Estimation of Platelia Dengue IgA Capture assay sensitivity in relation to DENV infecting serotype

The RT-PCR serotyping results obtained from the 134 dengue positive patients indicated that 46 (34.3%) patients were infected with DENV-1, 27 (20.1%) with DENV-2, 36 (26.9%) with DENV-3 and 25 (18.7%) patients were infected with DENV-4. The median (IQ1-IQ3) numbers of days after the onset of fever of DENV-1, DENV-2, DENV-3, DENV-4 sera were 7 (5–8), 8 (6–10), 10 (7–14), 7 (6–10), respectively (p<0.001). Comparisons between serotypes showed that DENV-3 sera were collected later than the DENV-1 and DENV- 4 serotypes.

The assessment results of the IgA test’s ability to detect positive sera in relation to DENV infecting serotype results are expressed in Table 3. The results show that the IgA index test is not as efficient in detecting infections of either serotype (p = 0.058). It had very good sensitivity for detecting infections caused by three dengue serotypes (DENV-2, DENV-3, DENV-4, with sensitivities of 100%, 97% and 92% respectively), while its ability to detect infection caused by DENV-1 was significantly less efficient (85%).

**Table 3:** IgA detection using Platelia Dengue IgA Capture assay in relation to DENV infecting serotype.

Dengue Serotype	No. of sera	No. of positive IgA	IgA positivity % (95% CI)
**DENV-1**	46	39	85 (71–94)
**DENV-2**	27	27	100 (87–100)
**DENV-3**	36	35	97 (85–100)
**DENV-4**	25	23	92 (74–99)

#### Estimation of Platelia Dengue IgA Capture assay sensitivity in relation to DENV immune status

Sera from DENV-infected patients were also tested for the presence of IgM and IgG using the Dengue IgM and Dengue IgG Capture ELISA (PanBio). Based on that results and according to the host immune status definition described above (see Material and methods), the immune status of 125 sera (93.3%) were identified among the 134 positive DENV patients (the nine others were unclassifiable according to the chosen criteria). Forty-two (33.6%) and 83 (66.4%) of these 125 classified sera were defined as primary or secondary infections, respectively.

The assessment results of the IgA test’s ability to detect positive sera in relation to immune status appear in Table 4. According to these estimated sensitivities, the IgA index test displayed a sensitivity of 100%, a better performance (p = 0.04) in detecting secondary dengue infections than primary infections. Out of the 42 primary positive sera, the IgA index assay detected 39 positive, indicating a sensitivity of 93% (95% CI, 81% to 99%).

**Table 4:** IgA detection using Platelia Dengue IgA Capture assay in relation to immune status.

Immune status	No. of sera	No. of positive IgA	IgA positivity % (95% CI)
**Primary**	42	39	93 (81–99)
**Secondary**	83	83	100 (96–100)

### Comparative analysis of the Platelia Dengue IgA Capture assay and the Panbio Dengue IgM Capture ELISA

In that study, the IgA index assay displayed a sensitivity of 93% (124/134; 95% CI, 87% to 96%), while the PanBio Dengue IgM kit positive for IgM detected 95% of the sera (127/134; 95% CI, 90% to 98%). These differences were not statistically different (p = 0.25). As observed for IgA, seropositivities for IgM also rose from 56% for sera collected 3–4 days after the onset of fever, to 100% for those collected five or more days after fever onset.

**Fig 1:**
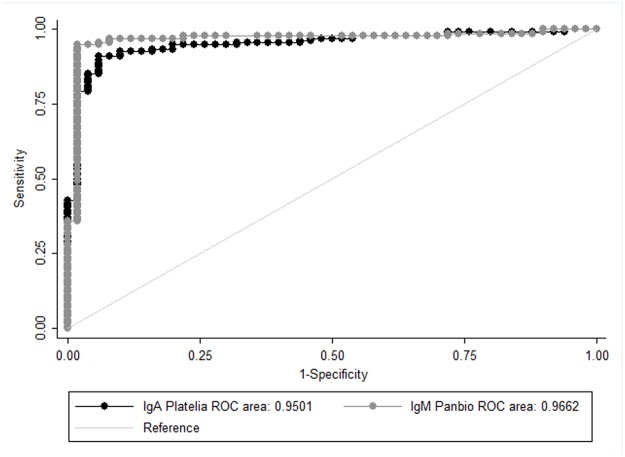
Platelia Dengue IgA Capture index test and Panbio Dengue IgM kit ROC curves.

Moreover, ROC curves were drawn for both IgA index test and Panbio IgM capture assay, both of which showed very good performances, with Area Under ROC around 0.95 (Fig. 1). The comparison between the two AUROC showed no difference (p = 0.4135). When using a qualitative approach, the results are similar with an almost perfect kappa coefficient, equal to 0.8632.

## Discussion

The novel Platelia Dengue IgA Capture assay’s performance is very good: the overall sensitivity and specificity of the IgA index test are 93% (124/134) and 88% (43/49), respectively, and only 0.54% (1/184) of the results was inconclusive. These results suggest that the Platelia Dengue IgA Capture assay is well correlated with dengue diagnosis on clinical sera from patients exhibiting a dengue-like syndrome. The development of this new assay could contribute to the improvement of dengue diagnostic performance, currently a major challenge in managing dengue disease [1,6]. Even if the detection of specific IgA in the serum has been evaluated as a useful diagnostic parameter [8,10,13,14], there is still no single available ELISA commercial kit to measure IgA antibodies. The only available Dengue IgA kit was recently developed by MP Diagnostic ASSURE as a rapid test (Dengue IgA RT), and even when evaluated under different conditions and inclusion criteria, the performances were lower, with an overall sensitivity of 61.0% and specificity of 85.1% by comparing the index test results with IgM detection by ELISA [22–24].

In concordance with theoretical IgA antibodies kinetics observed in serum, sensitivities of the Platelia Dengue IgA Capture index test vary according to the sera collection day. Sensitivity was highest when estimated on convalescent samples (day 8 to 15 collected sera), compared to acute samples (day 3 to 7 collected sera), displaying sensitivities of 100% for convalescent versus 84% (95% CI, 73% to 92%) for acute samples.

The effect of the collection day of sera data was also observed in the analysis concerning the infecting dengue virus serotype: performances obtained showed significantly lower accuracy in detecting serotype 1. To the best of our knowledge, no existing studies have reported such differences observed in a study based on anti-dengue immunoglobulin detections. Results of serological IgM test obtained on these samples suggest the same trend of better performance observed for serotype -2, -3 and -4 detection. Our dataset features, and particularly the small size of the groups, does not allow for more thorough statistical analysis of the specific effects of each of these variables however, this point could be fully explained by the differences observed between the collection day of sera data: matched to the median (IQ1-IQ3) numbers of days after the onset of fever, DENV-1 sera displayed the lowest value, i.e. 7(5–8), compared with 8 (6–10), 10 (7–14), 7 (6–10) for DENV-2, DENV-3, DENV-4 sera respectively.

The IgA test displayed good sensitivity in detecting immune status, with better efficiency at detecting secondary infections, when evaluated according to the IgM/IgG ratio method recommended by WHO. This test’s ability to detect IgA for primary and secondary dengue infections is a major criterion for quality and relevance, particularly in dengue (hyper)-endemic area where the detection of dengue secondary cases at early stage of infection is especially important, because secondary cases are more frequently associated with severe outcomes. This point should be correlated with collection day medians, which are lower for primary sera (7 (5–10) days), and higher for secondary ones (8 (6–11) days).

Moreover, the results leading to a specificity of 88% (6 sera found positive by the Platelia Dengue IgA Capture among the non-dengue group) could be correlated either with a previous dengue infection, preceding the recent dengue-like syndrome associated with these clinical samples, or with a cross-reaction potentially induced by another etiological agent, because of a higher risk of exposure to multiple flavivirus infections in French Guiana. A high degree of cross-reactivity is frequently observed among flavivirus infection serology, particularly where the circulation of multiple flaviviruses compromises the local specificity of such measurements.

In addition, it is interesting to note that global diagnostic performance reaches 100% for both specificity and sensitivity when adding the NS1 diagnosis assay. As expected, combining the NS1 assay with an IgA assay will enhance the sensitivity of detection. The estimated accuracy of this test evaluated under our criteria could provide better diagnostic value if combined with another direct diagnostic test, as commonly illustrated and recommended [1,4,6,7,25,26].

Finally, the comparable overall performances estimated for both IgM and IgA serological marker measurements make this IgA index test as reliable as the IgM test. Even if the most frequently used serological test is the IgM capture ELISA format, based on the fact that around 80% of patients are IgM positive by the 5th day following the onset of symptoms [1], its limitations from the anti-dengue IgM antibody persistence over several months make their detection can be due to infection up to several months earlier. They do not necessarily indicate an acute dengue infection. Because of longer IgM persistence in serum, IgA based method could be a more informative diagnosis tool because it is a marker of an earlier dengue infection, which narrows the time frame of infection and increases diagnostic accuracy [8,10–13].

To conclude, these results suggest that the Platelia Dengue IgA Capture assay is an acceptable test, with overall sensitivity and specificity of about 90%. This new test could contribute to dengue diagnosis, especially in countries where dengue virus is endemic and where many serotypes of dengue viruses are circulating. Using the IgA test assay to measure a good quality serological marker detectable in acute-phase serum and persisting for a shorter period of time than dengue specific IgM allows a more accurate dating of infection. It reduces the window of potential recent dengue infection and refines the diagnostic strategy for dengue adopted by physicians. Moreover, it would be interesting to increase the sample numbers per day after fever onset enough to minimize the effect of this variable on others. Lastly, it appears essential to design and to conduct prospective diagnostic evaluations during different phases of dengue epidemic in an endemic area [5,27].

## Supporting Information

S1 Checklist STARD checklist. (DOC)

S1 Flowchart Flowchart for the collection of sera from the French Guiana National Reference Center collection (Institut Pasteur). (PDF)
