# Supplementary material for: Evaluation of the Diagnostic Accuracy of a New Dengue IgA Capture Assay (Platelia Dengue IgA Capture, Bio-Rad) for Dengue Infection Detection
Source: PLoS Negl Trop Dis. 2015 Mar 24;9(3):e0003596. doi: 10.1371/journal.pntd.0003596 (PMC4372552; doi:10.1371/journal.pntd.0003596)
Supplement: S1 Flowchart — (PDF) [file pntd.0003596.s002.pdf]

**Study collection of sera from the French Guiana National Reference Center collection (Institut Pasteur) stored at -80°C**

Sera collected in French Guiana National Reference Center collection from patients exhibiting a dengue-like syndrome (fever, arthralgia, headache and/or myalgia) in French Guiana between 2002 and 2013 (n = 56766) :
-for diagnostic purposes
-for identifying the DENV serotype from patient sera already found positive for NS1 antigen in the context of epidemiological surveillance

**NRC DIAGNOSIS ANALYSIS**

**POSITIVE** serum samples for DENV infection (n = 48597)

**NEGATIVE** serum samples for DENV infection (n = 8169)

**SAMPLING FOR THIS STUDY**

**POSITIVE paired** serum samples (n = 4281)

**NEGATIVE paired** serum samples (n = 1282)

**POSITIVE** paired serum **samples sampled between the 3rd and the 15th day following the fever onset** (n = 736)

**NEGATIVE** paired serum samples **sampled between the 3rd and the 15th day following the fever onset** (n = 163)

**POSITIVE** acute-phase serum samples (n = 525) for either
- RT-PCR targeting viral RNA and/or
- viral isolation in *Aedes pseudoscutellaris* cell (AP61)

**NEGATIVE** for DENV infection (n = 105) if at least two of the three following analyses were obtained:
(i) Negative RT-PCR or viral isolation from samples collected on day 0 and day 5 of the disease
(ii) Negative NS1 detection of sera obtained prior to five days (Platelia™ Dengue NS1 AG, Bio-Rad)
(iii) Negative in-house IgM capture assay on no less than 8 days sera

**POSITIVE** serum samples with **sufficient volume** (n = 134)

**NEGATIVE** serum samples with **sufficient volume** (n = 50)

Dengue-infected patients group (134 sera)

Non-Dengue infected patients group (50 sera)

**PLATELIA™ DENGUE IGA CAPTURE ASSAY ASSESSMENT**

**Sensitivity : 124/134** **Specificity : 44/50** **Equivocal : 1/184**
